# Supplementary material for: Genetic Variants in Genes Related to Lung Function and Interstitial Lung Diseases Are Associated with Worse Outcomes in Severe COVID-19 and Lung Performance in the Post-COVID-19 Condition
Source: Int J Mol Sci. 2025 Feb 26;26(5):2046. doi: 10.3390/ijms26052046 (PMC11900979; doi:10.3390/ijms26052046)
Supplement: Supplementary file 1 [file ijms-26-02046-s001.zip › ijms-3465850-supplementary.pdf]

Supplementary material

Supplementary Table S1. Clinical and demographic data of patients with severe COVID-19 according to the invasive mechanical ventilation requirement.

Variable	IMV (n=726)	Non-IMV (n=210)	*p*-value
Age, years	59 (51-69)	56 (48-66)	**0.008**
Males, n (%)	515 (70.9)	134 (63.8)	0.050
BMI, kg/m^2	29.7 (26.2-33.7)	28.6 (25.9-32.8)	0.056
Hospital stay, days	23 (16-35)	11 (8-15)	**<0.001**
Symptoms onset, days	10 (7-14)	9 (7-12)	0.204
T2DM, n (%)	211 (29.1)	58 (27.6)	0.729
SAH, n (%)	254 (35.0)	68 (32.4)	0.510
Cardiopathies, n (%)	29 (4.0)	10 (4.8)	0.695
CRD, n (%)	66 (9.1)	16 (7.6)	0.580
Smoking, n (%)	213 (29.3)	52 (24.8)	0.223
TI, packs/years	4.9 (1.5-18.0)	5.1 (1.8-19.0)	0.585
Steroid administration n (%)	613 (84.4)	170 (80.9)	0.269
PaO_2/FiO_2 at hospital admission	98 (71-165)	195 (113-239)	**<0.001**
Deceased, n (%)	344 (47.4)	24 (11.4)	**<0.001**

Continuous data is presented as the median (interquartile range, Q1-Q3), and categorical data as the absolute value (frequency in percentage). The Mann-Whitney U and Exact Fisher's tests were used to compare the groups. BMI, body mass index; CRD, preexisting chronic respiratory diseases; IMV, invasive mechanical ventilation; SAH, systemic arterial hypertension; T2DM, type 2 diabetes mellitus; TI, tobacco index. The median of IMV days was 18 (11-28).

Supplementary Table S2. Genetic association study with invasive mechanical ventilation requirement in patients with severe COVID-19.

Gene/Genetic variant	Allele/ Genotype	IMV (n=726)	Non-IMV (n=210)	*p*-value	Adjusted *p*-value [a]
FAM13A		n=699	n=198		
rs2609255	T	956 (0.684)	272 (0.687)	0.909	0.924
	G	442 (0.316)	124 (0.313)		
	TT	326 (0.466)	94 (0.475)	0.965	0.873
	TG	304 (0.435)	84 (0.424)		0.748
	GG	69 (0.099)	20 (0.101)		
	Genetic association models				
	TT	326 (0.466)	94 (0.475)	0.835	0.942
	TG + GG	373 (0.534)	104 (0.525)		
	TT + TG	630 (0.901)	178 (0.899)	0.924	0.744
	GG	69 (0.099)	20 (0.101)		
TERT		n=396	n=149		
rs2736100	A	576 (0.727)	208 (0.698)	0.337	0.336
	C	216 (0.273)	90 (0.302)		
	AA	212 (0.535)	70 (0.470)	0.305	
	CA	152 (0.384)	68 (0.456)		0.137
	CC	32 (0.081)	11 (0.074)		0.936
	Genetic association models				
	AA	212 (0.535)	70 (0.470)	0.172	0.198
	CC + CA	184 (0.465)	79 (0.530)		
	CA + AA	364 (0.919)	138 (0.926)	0.787	0.653
	CC	32 (0.081)	11 (0.074)		
DSP		n=694	n=200		
rs2076295	T	806 (0.581)	228 (0.570)	0.703	0.614
	G	582 (0.419)	172 (0.430)		
	TT	232 (0.334)	69 (0.345)	0.466	0.997
	TG	342 (0.493)	90 (0.450)		0.397
	GG	120 (0.173)	41 (0.205)		
	Genetic association models				
	TT	232 (0.334)	69 (0.345)	0.778	0.435
	TG + GG	462 (0.666)	131 (0.655)		
	TT + TG	574 (0.827)	159 (0.795)	0.298	0.6
	GG	120 (0.173)	41 (0.205)		
TOLLIP		n=476	n=205		
rs111521887	C	916 (0.962)	395 (0.963)	0.913	0.986
	G	36 (0.038)	15 (0.034)		

	CC	440 (0.924)	191 (0.932)		
	CG	36 (0.076)	13 (0.063)	0.269	0.949
	GG	0 (0.000)	1 (0.005)		0.986
	Genetic association models				
	CC	440 (0.924)	191 (0.932)	0.736	0.844
	CG + GG	36 (0.076)	14 (0.068)		
	CC + CG	476 (1.000)	204 (0.995)	0.664	0.978
	GG	0 (0.000)	1 (0.005)		
THSD4		n=622	n=174		
rs872471	A	980 (0.788)	261 (0.750)	0.133	0.167
	G	264 (0.212)	87 (0.250)		
	AA	394 (0.633)	98 (0.563)		
	AG	192 (0.309)	65 (0.374)	0.232	0.092
	GG	36 (0.058)	11 (0.063)		0.831
	Genetic association models				
	AA	394 (0.633)	98 (0.563)	0.092	0.154
	AG + GG	228 (0.367)	76 (0.437)		
	AA + AG	586 (0.942)	163 (0.937)		
	GG	36 (0.058)	11 (0.063)	0.792	0.608

[a] Logistic regression model with adjustment for the co-variables age and PaO_2/FiO_2 levels at admission. IMV, invasive mechanical ventilation.

Supplementary Table S3. Allele and genotype frequencies in patients of the post-COVID-19 group.

Genetic variant	Allele/ Genotype	Post-COVID-19	Survivors (n=497)	*p*-value	Adjusted *p*-value [a]
FAM13A		**n=94**	**n=490**		
rs2609255	T	123 (0.654)	666 (0.680)	0.497	0.757
	G	65 (0.346)	314 (0.320)		
	TT	41 (0.436)	224 (0.457)	0.681	0.754
	TG	41 (0.436)	218 (0.445)		
	GG	12 (0.128)	48 (0.098)		
	Genetic association models				
	TT	41 (0.436)	224 (0.454)	0.708	0.884
	TG + GG	53 (0.520)	266 (0.546)		
	TT + TG	82 (0.872)	442 (0.902)	0.385	0.754
	GG	12 (0.128)	48 (0.098)		
TERT		**n=91**	**n=304**		
rs2736100	A	140 (0.769)	436 (0.717)	0.165	0.605
	C	42 (0.231)	172 (0.283)		
	AA	53 (0.582)	153 (0.503)	0.363	0.424
	CA	34 (0.374)	130 (0.428)		
	CC	4 (0.044)	21 (0.069)		
	Genetic association models				
	AA	53 (0.582)	153 (0.503)	0.185	0.765
	CC + CA	38 (0.418)	151 (0.497)		
	CA + AA	87 (0.956)	283 (0.931)	0.536	0.552
	CC	4 (0.044)	21 (0.069)		
DSP		**n=101**	**n=480**		
rs2076295	T	118 (0.584)	561 (0.584)	0.995	0.834
	G	84 (0.416)	399 (0.416)		
	TT	32 (0.317)	162 (0.337)	0.744	0.771
	TG	54 (0.535)	237 (0.494)		
	GG	15 (0.148)	81 (0.169)		
	Genetic association models				
	TT	32 (0.317)	162 (0.337)	0.689	0.954
	TG + GG	69 (0.683)	318 (0.663)		
	TT + TG	86 (0.851)	399 (0.831)	0.619	0.769
	GG	15 (0.148)	81 (0.169)		
TOLLIP		**n=100**	**n=471**		
rs111521887	C	195 (0.975)	911 (0.967)	0.720	0.657
	G	5 (0.025)	31 (0.033)		

	CC	95 (0.950)	441 (0.936)	0.813	0.884
	CG	5 (0.050)	29 (0.062)		
	GG	0 (0.000)	1 (0.002)		
	Genetic association models				
	CC	95 (0.950)	441 (0.936)	0.773	0.775
	CG + GG	5 (0.050)	30 (0.064)		
	CC + CG	100 (1.000)	470 (0.998)	1.000	0.999
	GG	0 (0.000)	1 (0.002)		
THSD4		**n=90**	**n=426**		
	A	141 (0.783)	655 (0.769)	0.673	0.397
	G	39 (0.217)	197 (0.231)		
	AA	59 (0.655)	259 (0.608)	0.436	0.664
	AG	23 (0.255)	137 (0.322)		
	GG	8 (0.088)	30 (0.070)		
rs872471	Genetic association models				
	AA	59 (0.655)	259 (0.608)	0.399	0.999
	AG + GG	31 (0.345)	167 (0.392)		
	AA + AG	82 (0.912)	396 (0.930)	0.542	0.624
	GG	8 (0.088)	30 (0.070)		

[a] Logistic regression model with adjustment for the co-variables sex, body mass index, days of hospital stay, chronic respiratory diseases, steroid administration, and invasive mechanical ventilation requirement during COVID-19 hospitalization.

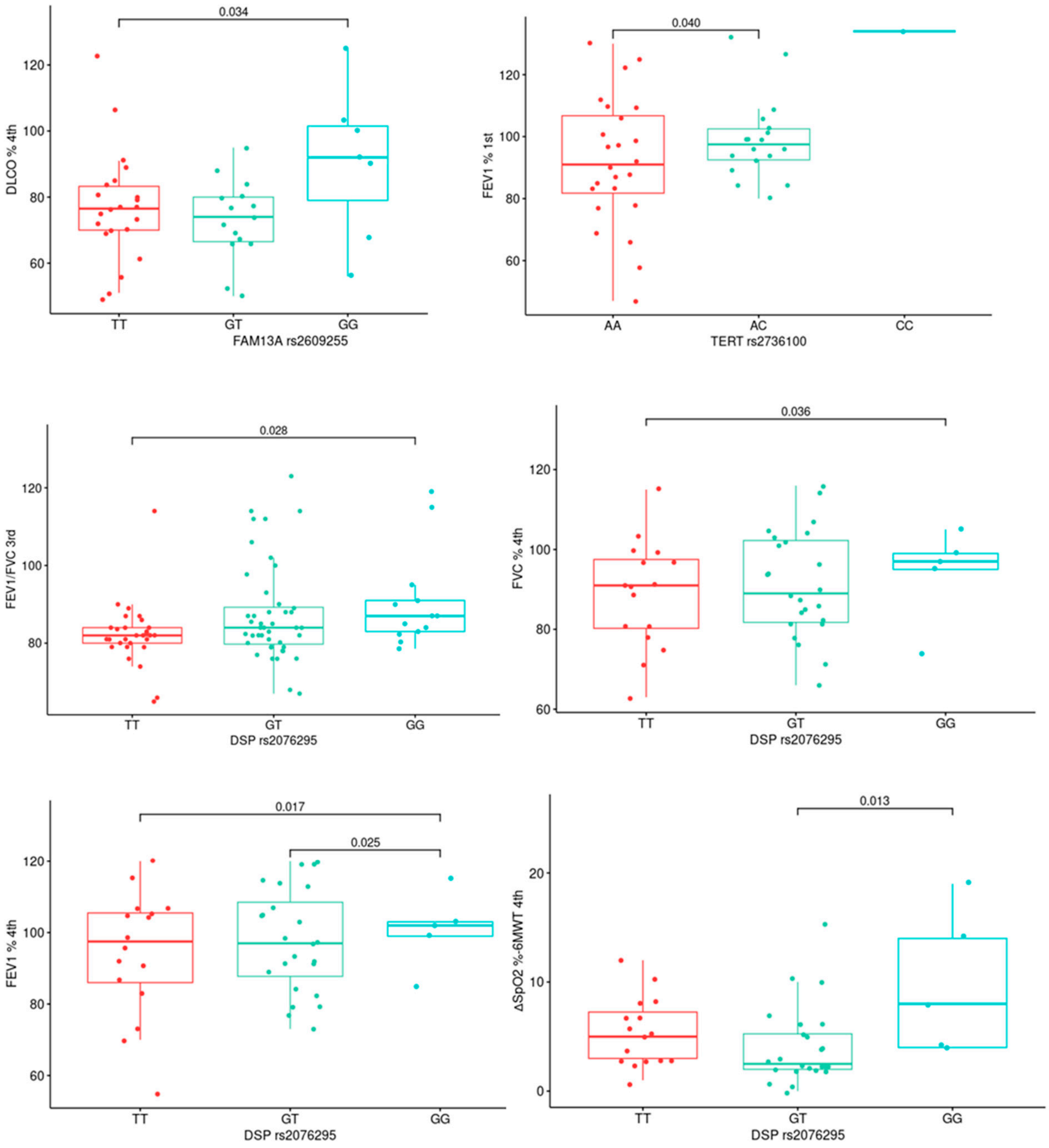

Supplementary Figure S1. Pulmonary function tests variation according to the genotypes of *FAM13A* rs2609255, *TERT* rs2736100, and *DSP* rs2076295 variants. Only results with statistically significant p-values (<0.05) are presented. FEV_1 forced expiratory volume in the first second; FVC forced vital capacity; DL_{CO}, diffusing capacity of the lungs for carbon monoxide; 6MWT, six-minute walk test; ΔSpO_2, delta of oxygen saturation determination at onset and at the end of the performance of the 6MWT.

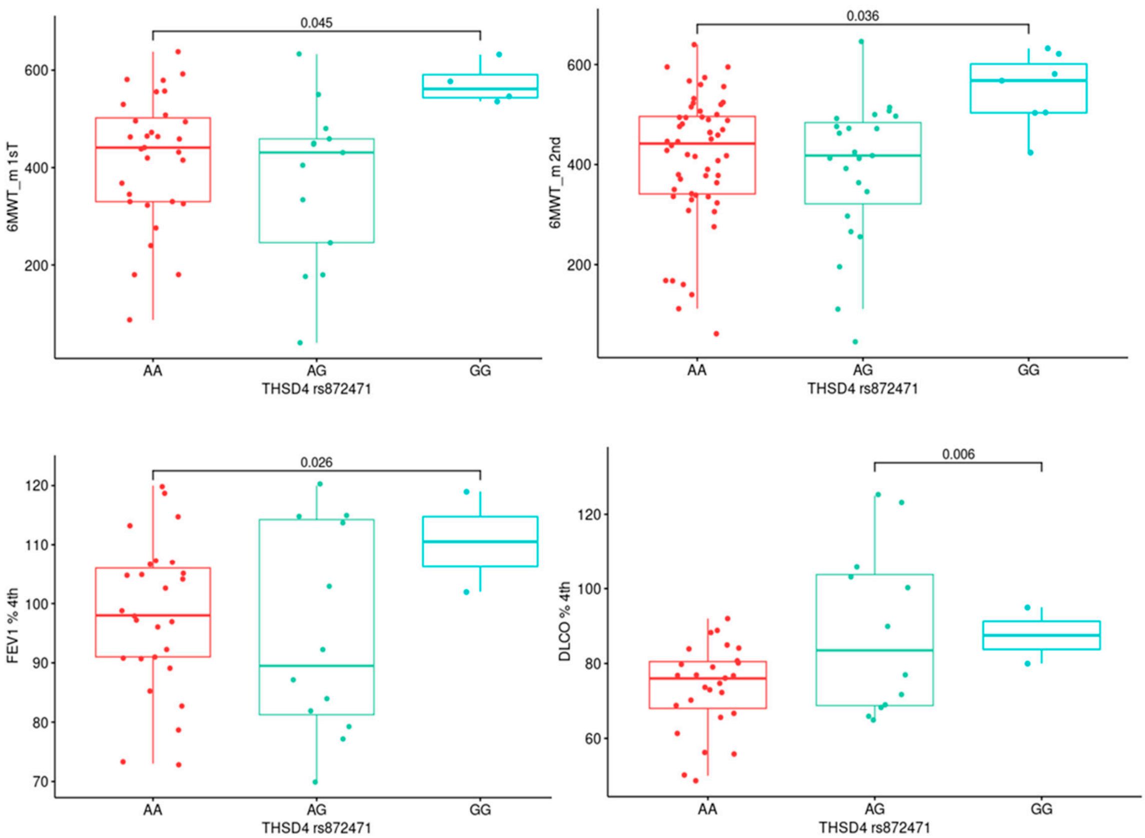

Supplementary Figure S2. Pulmonary function tests variation according to the genotypes of the *THSD4* rs872471 variant. Only results with statistically significant p-values (<0.05) are presented. FEV_1 forced expiratory volume in the first second; FVC forced vital capacity; DL_{CO}, diffusing capacity of the lungs for carbon monoxide; 6MWT, six-minute walk test; ΔSpO_2, delta of oxygen saturation determination at onset and at the end of the performance of the 6MWT.

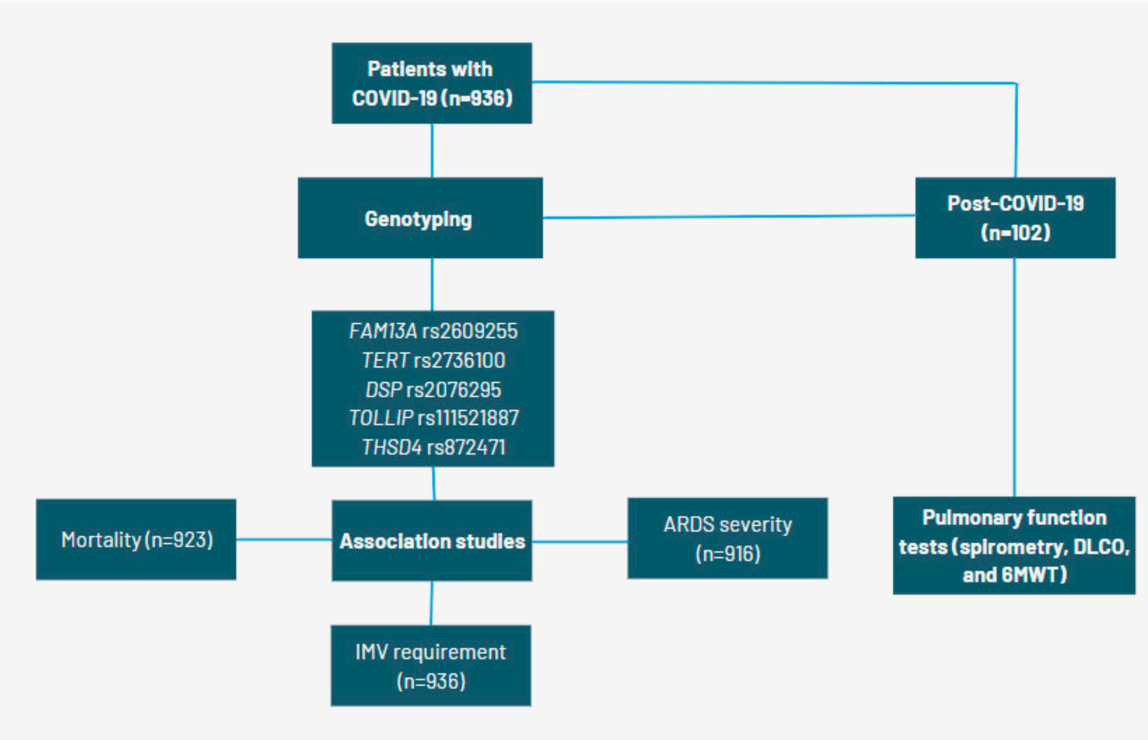

Supplementary Figure S3. Flow chart of the genetics association studies performed. The association studies only included the subjects in which the data of clinical outcome (mortality, IMV requirement, ARDS severity) was available. ARDS, acute respiratory distress syndrome; DLCO, diffusing capacity of the lungs for carbon monoxide; IMV, invasive mechanical ventilation; 6MWT, six-minute walk test.
